# Bone Metabolism Markers and Bone Mineral Density in Patients on Long-Term Acenocoumarol Treatment: A Cross-Sectional Study

**DOI:** 10.3390/jcm7100372

**Published:** 2018-10-20

**Authors:** Jolanta Sawicka-Powierza, Ewa Jablonska, Wioletta Ratajczak-Wrona, Dorota Rogowska-Szadkowska, Marzena Garley, Alicja M. Oltarzewska, Slawomir Chlabicz, Jerzy Konstantynowicz

**Affiliations:** 1 Department of Family Medicine, Medical University of Bialystok, Bialystok 15-054, Poland; dszadkowska@bsk.vectranet.pl (D.R.-S.); amoltarz@gmail.com (A.M.O.); schlabicz@poczta.onet.pl (S.C.); 2 Department of Haematology, Medical University of Bialystok, Bialystok 15-276, Poland; 3 Department of Immunology, Medical University of Bialystok, Bialystok 15-269, Poland; ewa.jablonska@umb.edu.pl (E.J.); rwioletta@umb.edu.pl (W.R.-W.); marzena.garley@umb.edu.pl (M.G.); 4 Department of Pediatric Rheumatology, Immunology, and Metabolic Bone Diseases, Medical University of Bialystok, Bialystok 15-274, Poland; jurekonstant@o2.pl

**Keywords:** acenocoumarol, bone mineral density, bone metabolism, osteocalcin, vitamin K antagonists

## Abstract

The aim of this study was to evaluate levels of osteocalcin (OC), osteoprotegerin (OPG) and total soluble receptor activator of nuclear factor-κB ligand (RANKL), and bone mineral density (BMD) in patients on long-term acenocoumarol (AC) treatment. The cross-sectional study was carried out in 42 patients treated long-term with AC and 28 control subjects. Serum concentrations of OC, OPG, and sRANKL were measured using enzyme linked immunosorbent assay (ELISA) kits, and BMD at the femoral neck and lumbar spine were assessed by dual energy X-ray absorptiometry. A significantly decreased concentration of OC was found in AC users compared to control subjects (4.94 ± 2.22 vs. 10.68 ± 4.5; *p* < 0.001). Levels of OPG, sRANKL logarithm (log), sRANKL/OPG log ratio, and BMD were comparable between. In female AC users, positive correlations between OC and RANKL log, and between OC and RANKL/OPG log ratio (*p* = 0.017; *p* = 0.005, respectively), and a negative correlation between OC and OPG (*p* = 0.027) were found. Long-term AC anticoagulation significantly decreases OC concentration, but does not affect other bone metabolism markers or BMD. Our results also suggest the possibility that long-term treatment with AC may alleviate bone resorption in postmenopausal women.

## 1. Introduction

Bone metabolism is characterized by two opposite actions: formation of new bone by osteoblasts and degradation (resorption) of old bone by osteoclasts. Osteocalcin (OC), a non-collagenous hydroxyapatite binding protein produced mainly by osteoblasts, is the only matrix protein synthesized exclusively in bone [1,2] and has a regulatory role in bone mineralization [3].

Vitamin K is a coenzyme for glutamate carboxylase, aimed at mediating the conversion of glutamate (Glu) to γ–carboxyglutamate (Gla), which is essential for full functionality of OC, enabling it to bind calcium [4,5]. Vitamin K antagonists (VKAs), including warfarin and acenocoumarol (AC), affect OC carboxylation through the inhibition of the vitamin K-reductase [6], subsequently leading to the production of the functionally inactive form of OC and thus disturb bone metabolism [7]. 

Recently, it has been shown that molecules belonging to the tumor necrosis factor (TNF) superfamily, such as receptor activator of nuclear factor-κB (RANK), receptor activator of nuclear factor- κB ligand (RANKL), and osteoprotegerin (OPG), are critical in the final stage of formation, differentiation and activation of osteoclasts. The effects of cytokines and hormones on bone metabolism have already been found to be associated with a change in the RANKL/OPG ratio. RANKL, produced by the osteoblastic cell line and activated T cells, is the factor that activates the formation process of mature osteoclasts by connecting with the RANK [8,9]. On the contrary, the biological effect of OPG, produced by various cells, including osteoblasts [10,11,12], is oppositional to the effects modulated by RANKL. OPG is its decoy soluble receptor that prevents binding of RANKL to RANK [13,14]. Thus, RANKL is responsible for bone resorption, while OPG stops the entire osteoclast maturation pathway, and inhibits resorption. Under physiological conditions RANKL and OPG are in balance. In the case where RANKL is more prevalent than OPG, the rate of resorption is pathologically increased, whereas in the case of OPG predominance over RANKL, the rate of bone resorption is pathologically reduced [15].

There are only few reports available that focus on bone metabolism markers in patients treated with VKAs, and the results are conflicting. Therefore, this study was carried out to evaluate levels of OC, OPG and total soluble RANKL, and bone mineral density (BMD) in patients on long-term AC treatment. 

## 2. Materials and Methods

### 2.1. Study Population

The cross-sectional study was carried out among adult patients on long-term AC treatment and healthy controls, who were recruited from the population of a primary care practice [16]. Participants’ data was initially retrieved from an electronic database and medical records. An age- and gender-matched control group was recruited from the healthy population using a random numbers table. The final qualification of all participants was based on medical records and a questionnaire containing questions about chronic diseases, medications and dietary supplements used, time of menopause (if applicable), smoking habits (past and current), and eating habits. The inclusion criteria consisted of written informed consent and the duration of AC treatment longer than 3 months.

Fifty patients and 50 controls were recruited into the study. Eight individuals receiving AC, as well as eight controls, did not meet eligibility criteria and were excluded. Additionally, 14 healthy subjects failed to adhere to the dual-energy X-ray absorptiometry (DXA) scan. Finally, 42 Caucasian individuals (20 females, 22 males) receiving long-term AC treatment for recurrent venous thromboembolism (*n* = 16; 38.1%), atrial fibrillation (*n* = 11; 26.2%) or mechanical heart valve prostheses (*n* = 15; 35.7%) were enrolled into the study. The patients receiving AC were monitored by international normalized ratio (INR) measurements and the mean INR was 2.45 ± 0.66. The mean duration of AC treatment was 10.02 ± 5.1 years. Twenty-eight healthy age- and gender-matched subjects (14 females, 14 males) constituted the control group.

Participants with chronic diseases that interfere with bone metabolism (i.e., chronic renal, liver, endocrine, connective tissue, metabolic diseases, depression, or prolonged immobilization), those receiving treatment, affecting bone and mineral metabolism (i.e., bisphosphonates, anticonvulsants, systemic glucocorticosteroids, loop and thiazide diuretics, thyroid hormones, estrogen, vitamin D and/or K2 supplements and calcium), and also vegetarians and vegans were excluded from the study. None of the participants had any conditions that would have made ambulating difficult and the majority were active people working in their profession.

The study protocol and procedures were approved by the Ethics Committee of the Medical University of Bialystok (No R-I-002/88/2013).

### 2.2. Measurements

Blood samples from all participants were collected after overnight fasting, and were centrifuged immediately, and sera were stored at −70 °C until measurement. 

Bone metabolism markers, OC, OPG and sRANKL, were measured using the ELISA method. The OC concentration in serum was assessed by sandwich enzyme linked immunosorbent assay (ELISA) using a commercially available kit (product specification KAQ1381, Invitrogen^TM^, Camarillo, CA, USA). OPG concentration was detected using an ELISA kit (product specification ab100617, Abcam, Cambridge, UK). Total soluble RANKL concentration was determined using an ELISA kit (product specification K1016, Immundiagnostik AG, Bensheim, Germany). All ELISA kits were used following the manufacturer’s instructions. Sensitivities of the assays were as follows: 0.08 ng/mL (OC), 1 pg/mL (OPG), and 1.56 pg/mL (sRANKL). Values of intra- and inter-assay coefficients of variation of ELISA tests turned out be: 4.5% and 3.5% for OC, <10% and <12% for OPG, and 0.9% and 9.3% for sRANKL, respectively. Absorbance was measured at 450 nm using an UVN-340 ASYS Hitech GmbH microplate reader (Biogenet, Eugendorf, Austria). 

Standing height and body weight were measured using standard anthropometric methods (wall-mounted stadiometer, electronic scale; Seca, Germany) and body mass index (BMI) was calculated using the standard formula.

Bone mineral density was determined at the femoral neck and lumbar spine (vertebrae L1–L4) by dual-energy X-ray absorptiometry (DXA), using Lunar Prodigy equipment (GE-Healthcare). BMD values were reported using crude values (g/cm^2^) and expressed as T-scores and Z-scores, based on appropriate reference data. Quality assurance and standard calibrations were carried out according to the manufacturer’s recommendations, and the reproducibility error based on the coefficient of variance was 1.9% for the lumbar spine. Scans and analyses of DXA were performed by a certified clinical densitometrist.

### 2.3. Statistical Methods

Statistical analyses were performed with the use of statistica 13. Qualitative data were presented in cross tables containing numbers and percentages. Quantitative data were presented as mean, minimum, maximum and standard deviation. Due to the quite profound positive asymmetry of sRANKL concentrations expressed in pg/mL and sRANKL/OPG ratios, a logarithmic transformation was used, after which distributions of these parameters were close to normal. The levels of analyzed parameters were compared using the Student’s *t*-test. Interdependence between the pairs of qualitative or categorized variables was compared using Pearson’s chi-squared test. The strength of interdependence between pairs of measurable parameters was measured using Pearson’s linear correlation coefficient, and statistical significance was measured using Student’s *t*-test for the correlation coefficient. The combined influence of gender and AC use on the level of the examined variables was evaluated by the two-way analysis of variance (ANOVA). The Fisher’s least significant difference (LSD) post hoc test was used to assess the significance of differences between pairs of subgroups. Differences in the levels of the analyzed parameters and correlations were considered statistically significant at *p* < 0.05.

## 3. Results

No differences in terms of age, gender, BMI, time elapsed since menopause (females), and smoking habits were found between AC users and the control group. The sRANKL concentration, and sRANKL/OPG ratio did not demonstrate normal distribution. The median (quartiles) of sRANKL concentration, and sRANKL/OPG ratio in AC users and controls were 66,957 pg/mL (23,200.4; 161,851) vs. 44,441.4 pg/mL (16,353.7; 156,897.2) and 2520.7 (944.1; 7306.4) vs. 1866.7 (625; 6559.3), respectively. After logarithmic transformation, distributions of these variables were close to normal. The mean concentration of OC was significantly decreased in AC users compared to controls. The mean OPG concentration, sRANKL log, sRANKL/OPG log ratio, and BMD values were comparable in both groups (Table 1). 

Anthropometric, biochemical parameters and BMD at the femoral neck and lumbar spine did not significantly differ between the groups of patients receiving AC prophylaxis for recurrent venous thromboembolism, atrial fibrillation and mechanical heart valve prostheses.

The Fisher’s LSD method used in analysis of ANOVA showed, that mean concentrations of OC in female and male AC users were significantly decreased compared to the gender-matched control groups (*p* < 0.001). There were no differences in OPG concentration, sRANKL log, sRANKL/OPG log ratio, and BMD values at the lumbar spine and femoral neck between female AC users and female controls, nor between male AC users and male controls either (Table 2).

**Figure 1:**
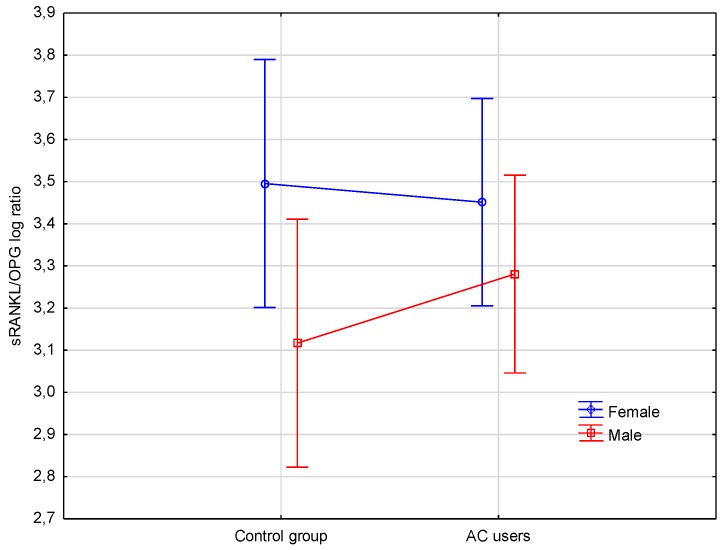
Levels of sRANKL/OPG log ratio in female and male controls, and female and male AC users. sRANKL, soluble receptor activator of nuclear factor-κB ligand; log, logarithm; AC, acenocoumarol; OPG, osteoprotegerin.

The influence of two independent factors (gender and AC use) on the level of sRANKL/OPG (dependent variable) log ratio is shown on Figure 1. It was found, that AC use did not significantly affect the level of sRANKL/OPG log ratio (*p* < 0.658), while the level of sRANKL/OPG log ratio was higher in both female groups (AC users and controls) in comparison to both male groups (*p* < 0.045). When compared with the Fisher’s LSD test, the RANKL/OPG log ratio had a trend to increase on the border of significance in female controls compared to male controls (*p* < 0.074) (Table 2). 

In AC users, a positive correlation between concentration of OC and sRANKL log, and between OC and sRANKL/OPG log ratio was observed (*r* = 0.354, *p* = 0.021; *r* = 0.342, *p* = 0.027, respectively). sRANKL log correlated positively with sRANKL/OPG log ratio (*r* = 0.983, *p* < 0.001). BMD at femoral neck, T-score and Z-score correlated negatively with age (*r* = −0.672, *p* < 0.001; *r* = −0.723, *p* < 0.001; *r* = −0.543, *p* < 0.001, respectively). BMD at lumbar spine and T-score correlated negatively with age (*r* = −0.366, *p* = 0.017; *r* = −0.401, *p* = 0.009, respectively), while Z-score correlated negatively with BMI (*r* = −0.334, *p* = 0.03). 

**Figure 2:**
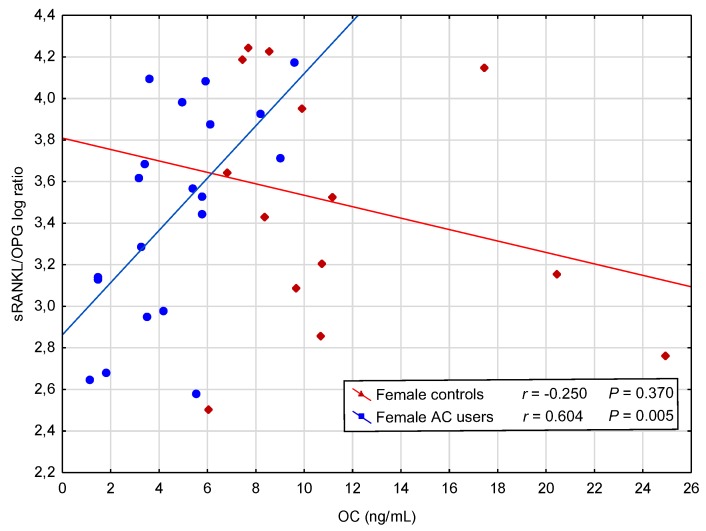
A positive correlation between concentration of osteocalcin (OC) and soluble receptor activator of nuclear factor-κB ligand (sRANKL)/osteoprotegerin (OPG) logarithm (log) ratio in female acenocoumarol (AC) users.

However, further analysis showed positive correlations in female AC users between OC and RANKL log, and between OC and RANKL/OPG log ratio (Figure 2), and a negative correlation between OC and OPG. In contrast, in male AC users, a positive correlation between concentration of OC and OPG was observed (Table 3). 

## 4. Discussion

This study showed that osteocalcin (OC) is strongly affected by oral anticoagulant treatment. The significantly decreased concentration of OC observed in men and women on long-term AC treatment may indicate a negative effect of this therapy on OC status and possibly on bone metabolism, by disrupting bone formation and osteoid mineralization. OC knockout mice developed a phenotype marked by higher bone mass [17]. Convincing results were obtained in a study on rats, demonstrating that OC plays a key role in the formation of properly mineralized nodules in the bone. Immunoelectron microscopy revealed lower OC content in the warfarin-administered osteoid, which featured scattered crystalline particles [18]. Thus, OC plays a key role in the formation of mineralized bone with proper spatial structure of mineral crystals. The dispersed mineral crystals could presumably lead to reduced bone strength and an increased fracture risk in patients treated with VKAs, despite the normal BMD. Several studies have investigated whether patients treated with VKA were at an increased risk of bone fractures, but the results are conflicting. A meta-analysis, based on large data from 21 studies, found no association between VKA treatment and fracture risk [19]. Furthermore, in a recent study, Steffel et al. [20] reported no differences in the risk of fractures between patients treated with edoxaban and warfarin. VKA-induced deterioration of cortical bone material quality, due to reduced OC concentration, may be compensated for by adaptation of cortical bone structure, thus not leading to increased fracture risk. In turn, Lau et al. reported a lower risk of fragility fractures in patients treated with dabigatran in comparison to those treated with warfarin [21]. Therefore, the evidence regarding increased risk of fractures in patients on VKA remains questionable, and probably needs further investigation in larger prospective cohorts. Furthermore, OC inhibits the precipitation of hydroxyapatite from supersaturated solutions of calcium and phosphate [4,22] and may be how excessive mineralization of the growth plate is normally prevented in vivo. Our assumptions are in line with the results of a study by Price et al. in rats, where excessive mineralization of the growth plate, following 8-month-long warfarin administration, was confirmed [23]. Therefore, it can be supposed, that OC deficiency in patients on long-term AC treatment may contribute to excessive bone mineralization with scattered crystalline particles in osteoid. Densitometry is not able to determine abnormalities in bone microarchitecture or quality of bone, and abnormal distribution of crystalline particles may be imperceptible in the DXA scans. It is possible, that a long-term disturbance of bone formation associated with low OC levels in AC users may have a detrimental effect on bone structure and quality, and therefore, result in the formation of bone with a greater risk of being fragile. However, it is difficult to draw such a definite conclusion using a cross-sectional design.

There were no significant changes neither in femoral nor lumbar BMD between AC users and control subjects, irrespective of adjustment used or gender. Systematic review and meta-analysis of 21 studies, including 79,663 individuals treated with VKA vs. 597,348 controls, showed similar BMD values at all investigated sites. Only a single study showed significantly lower spine T-scores in the VKA-treated patients [19]. A prospective study on male rhesus monkeys, that received warfarin for 3 years, also confirmed no changes in both, markers of skeletal turnover, or BMD in male primates [24].

Our results are consistent, to a great extent, with those obtained by other researchers who reported both, a decreased OC concentration [25,26], and a decreased OC concentration without significant bone loss [27,28] in patients treated with VKA. Some animal studies have also showed a decreased concentration of OC as a result of warfarin administration [23,29,30]. In turn, other authors showed a concurrent decrease in the concentration of OC along with bone loss in patients using VKA [31,32,33]. On the contrary, one study revealed no change in OC concentration, while a reduction in BMD was demonstrated in patients treated with VKA [34]. Different recruitment methods, designs or methodologies, the influence of still unknown factors, different duration of treatment or comorbidities may have caused such differences in results across studies presented above.

In our study, we did not observe significant differences in concentration of OPG, sRANKL, and sRANKL/OPG ratio between AC users and controls, nor between male and female AC users and sex-matched controls. In our opinion, the decreased OC synthesis in AC users cannot be regarded as a result of impaired osteoblast activity, because the use of AC did not affect other bone markers. Our data do not rule out, however, that the quality of bone tissue is negatively affected by the production of inactive undercarboxylated OC. On this basis, we conclude that the use of AC does not affect other bone metabolism markers; therefore, the rate of bone loss remains unchanged. The information available in literature on bone metabolism in patients treated with VKAs is scarce and inconsistent. Similarly, Knapen et al. [26] found, in a randomized longitudinal study, that bone turnover in patients treated with VKA remained unchanged. Such a conclusion was drawn from the fact that, with the exception of OC, oral anticoagulant therapy had no effect on markers of osteoblast or osteoclast function, which is indicative of an unchanged rate of bone loss. In contrary, a recent study by Namba et al. showed a significantly higher concentration of RANKL in patients treated with warfarin, mainly in men (22 males and 2 females), and based on this, they concluded that warfarin therapy might be related to bone mineral loss [35]. In contrast, decreased rate of bone turnover in men treated with AC was shown by Pluskiewicz et al. [36], their conclusion being drawn from the significantly decreased concentrations of bone alkaline phosphatase, the marker of bone formation, and the C-terminal telopeptide of type I collagen, the marker of bone resorption, accompanied by unaffected BMD in bone densitometry measurements. Stenova et al. [28] also demonstrated both, significantly decreased serum OC concentration and C-terminal telopeptide of type I collagen concentration in coumarin-treated patients compared to healthy controls. Based on their findings, the authors stated that vitamin K has an influence on bone turnover. The discrepancy between results obtained by us and other authors may be due to various durations of therapy, a diversified grade of OC suppression, and/or skeleton condition prior to the initiation of VKA treatment. Such divergent results may also derive from the fact that some studies were carried out on a population of patients with pre-existing morbidity or with other factors influencing bone metabolism, e.g., vitamin D deficiency. It is already known that both vitamins, D and K2, are responsible for the synthesis of the active form of OC, which may be also modulated by vitamin D deficiency [37].

It was found that the use of AC did not significantly affect the levels of sRANKL/OPG ratios in female and male AC users. However, the level of sRANKL/OPG ratio was significantly higher in both female groups (AC users and controls) in comparison to both male groups, and it was greater in female controls compared to male controls. To the best of our knowledge, our study is the first to demonstrate positive correlations between concentration of OC and sRANKL and between OC and sRANKL/OPG ratio, and also a negative correlation between concentration of OC and OPG in female AC users. The process of bone resorption may continue until the compensatory mechanism preventing bone resorption, detectable as an OPG increase, is activated. It was reported that ovariectomy-induced bone loss might be enhanced in OC deficient mice [4]. Our findings, however, indicate the possibility that long-term treatment with AC may alleviate bone resorption in postmenopausal women. The mechanisms may be sex-specific, as such a compensatory mechanism was not noted in male AC users. These differences between women and men may have been associated with the fact, that most of the women in our study were over 50 years old and may have had physiologically increased bone resorption due to menopausal deficiency of sex hormones.

The major limitations of our study were, firstly, the small sample size, and secondly, the observational design of the study, which did not allow us to draw firm conclusions about causality. Furthermore, the inability to assess the concentration of undercarboxylated OC and also the lack of bone turnover markers in this study may limit a spectrum of possible associations.

In summary, the results of our study reveal an association between long-term AC treatment and decreased OC concentration, however without significant changes in markers of bone metabolism, and, importantly, without a decrease in bone mineral density. Our results also suggest the possibility that long-term treatment with AC may alleviate bone resorption in postmenopausal women. Therefore, the concentration of OC in combination with RANKL/OPG ratio appear to be a more important marker of bone metabolism in postmenopausal female patients treated with AC, than OPG or RANKL alone. Further prospective investigations are needed to address the question whether chronic suppression of OC in patients treated persistently with AC affects bone quality, and/or increases the risk of fragility fractures. Considering the fact that anticoagulant therapy with VKA is still widely used, possible adversity of this treatment on bone health appears important in clinical practice.

## Figures and Tables

**Table 1:** Patients’ characteristics, biochemical parameters and BMD values in the study subjects.

Characteristics	AC Users (*n* = 42)	Control Subjects (*n* = 28)	*p* Value
Gender (male/female), *n* (%)	22 (52.4)/20 (47.6)	14 (50)/14 (50)	0.845
Smokers/non-smokers, *n* (%)	6 (14.3)/36 (83.7)	3 (10.7)/25 (89.3)	0.662
Age, years	64.67 ± 8.74 (40–81)	63.29 ± 9.8 (39–84)	0.54
BMI, kg/m^2^	28.82 ± 5.33 (16.9–41.09)	26.76 ± 3.6 (19.8–34.2)	0.078
Time post-menopause, years	14.05 ± 8.38 (0–28)	13 ± 11.37 (0–34)	0.758
Osteocalcin (OC), ng/mL	4.94 ± 2.22 (1.16–11.8)	10.68 ± 4.5 (6.03–25)	<0.001
Osteoprotegerin (OPG), pg/mL	27.53 ± 7.1 (17.93–54.6)	25.42 ± 5.5 (14.93–36.4)	0.19
sRANKL log	4.79 ± 0.53 (3.75–5.63)	4.67 ± 0.58 (3.75–5.63)	0.51
sRANKL/OPG log ratio	3.36 ± 0.54 (2.22–4.29)	3.31 ± 0.58 (2.32–4.2)	0.685
Femur neck BMD, g/cm^2^	0.93 ± 0.16 (0.64–1.31)	0.91 ± 0.1 (0.69–1.2)	0.6
Femur neck T-score	−0.77 ± 1.28 (−2.86–2.75)	−0.85 ± 1.1 (−2.71–1.6)	0.78
Femur neck Z-score	0.15 ± 1.07 (−1.61–3.09)	0.1 ± 1.0 (−2.06–2)	0.845
Lumbar spine BMD (L1–L4), g/cm^2^	1.12 ± 0.17 (0.78–1.53)	1.06 ± 0.17 (0.73–1.4)	0.091
Lumbar spine BMD T-score	−0.66 ± 1.38 (−3.32–2.61)	−1.25 ± 1.4 (−3.72–1.8)	0.085
Lumbar spine BMD Z-score	−0.12 ± 1.22 (−2.66–2.42)	−0.53 ± 1.2 (−3.32–1.4)	0.18

AC, acenocoumarol; BMD, bone mineral density; BMI, body mass index; log, logarithm; sRANKL, soluble receptor activator of nuclear factor-κB ligand. Data are shown as a number (percentage) or Mean ± SD (range); A *p* value of < 0.05 is considered statistically significant.

**Table 2:** The influence of gender and the use of AC on the level of the examined variables in female and male AC users and controls.

Variables	Female AC Users	Female Controls	Male AC Users	Male Controls
Number	20	14	22	14
OC, ng/mL	4.68 ± 2.44 ^a^	11.42 ± 5.58	5.17 ± 2.03 ^b^	9.94 ± 2.98
OPG, pg/mL	27.81 ± 7.03	24.12 ± 6.26	27.27 ± 7.38	26.72 ± 4.42
sRANKL log	4.88 ± 0.48	4.86 ± 0.58	4.71 ± 0.58	4.54 ± 0.52
sRANKL/OPG log ratio	3.45 ± 0.51	3.5 ± 0.59	3.28 ± 0.58	3.12 ± 0.53
Femur neck BMD, g/cm^2^	0.91 ± 0.18	0.86 ± 0.11	0.95 ± 0.14	0.96 ± 0.11
Lumbar spine BMD (L1–L4), g/cm^2^	1.07 ± 0.17	1.0 ± 0.16	1.17 ± 0.16	1.1 ± 0.17

AC, acenocoumarol; OC, osteocalcin; OPG, osteoprotegerin; log, logarithm; sRANKL, soluble receptor activator of nuclear factor-κB ligand; BMD, bone mineral density. Data are shown as a Mean ± SD; Female AC users vs. Female controls ^a^ *p* < 0.001; Male AC users vs. Male controls ^b^ *p* < 0.001.

**Table 3:** Correlations between studied variables in female and male AC users, and gender-matched controls.

Variables		Values of Pearson’s Linear Correlation Coefficients							
		Female AC Users *n* = 20		Female Controls *n* = 14		Male AC Users *n* = 22		Male Controls *n* = 14	
		*r*	*p*	*r*	*p*	*r*	*p*	*r*	*p*
OC, ng/mL	OPG (pg/mL)	−0.495	0.027	−0.338	0.237	0.701	<0.001	0.095	0.747
	sRANKL log	0.528	0.017	−0.33	0.25	0.258	0.247	−0.15	0.609
	sRANKL/OPG log ratio	0.604	0.005	−0.25	0.37	0.148	0.513	−0.161	0.582

AC, acenocoumarol; OC, osteocalcin; OPG, osteoprotegerin; log, logarithm; sRANKL, soluble receptor activator of nuclear factor-κB ligand.
